# “Atypical psychoses” and anti‐NMDA receptor encephalitis: A review of literature in the mid‐twentieth century

**DOI:** 10.1111/pcn.13317

**Published:** 2021-12-23

**Authors:** Tomoko Komagamine, Takashi Kanbayashi, Keisuke Suzuki, Koichi Hirata, Seiji Nishino

**Affiliations:** ^1^ Department of Neurology Dokkyo Medical University Tochigi Japan; ^2^ International Institute for Integrative Sleep Medicine University of Tsukuba Ibaraki Japan; ^3^ Ibaraki Prefectural Medical Center of Psychiatry Ibaraki Japan; ^4^ Department of Psychiatry and Behavioral Sciences Stanford University School of Medicine Stanford California USA

The concept of “atypical psychoses” was proposed in the twentieth century under the influence of “the theory of localization of brain function” by Wernicke in opposition to Kraepelin's “two‐entities principle” of schizophrenia and bipolar disorder. Atypical psychoses have been given different names, including cycloid psychosis, acute schizoaffective psychosis, postpartum psychosis, bouffée délirante, and reactive psychosis. In Japan, Hisatoshi Mitsuda assumed the heterogeneity of schizophrenia and proposed subgroups,^1^ termed an atypical group “atypical psychoses”. The term was later merged with an international consensus and expanded to a more comprehensive concept (Table S1). In the Diagnostic and Statistical Manual of Mental Disorders (DSM)‐III, the interpretation of “atypical psychoses” was expanded into a residual category for unclassified mental disorder, which disappeared after the DSM‐IV.

Among Japanese psychiatrists, common features of atypical psychoses were recognized as follows: acute onset, a monophasic or polyphasic course without sequelae, and impaired consciousness and emotion with psychomotor disturbances.^2^ Fever, potentially lethal dysautonomia, and the higher prevalence in women were also considered characteristics of atypical psychoses. Abnormal electroencephalograms, including dysrhythmia and slow wave bursts, were considered evidence for the diagnosis of atypical psychoses in patients with acute psychosis.^3^ Corticosteroid and electroconvulsive therapy were sometimes used and found to be effective in treating atypical psychoses.^4^, ^5^ These clinical characteristics resemble the modern entity of antibody‐mediated encephalitis, especially anti‐*N*‐methyl‐D‐aspartate receptor (NMDAR) encephalitis (Table S2).

We reviewed case series of Mitsuda's “atypical group” published in 1942^1^ and Stauder's “lethal catatonia syndrome”, which was considered the most severe form of atypical psychoses at that time, published in 1934^6^ and analyzed them in the context of modern anti‐NMDAR encephalitis. Detailed descriptions of the review and the discussions are provided in the Supplementary Information.

The “atypical group” was compared with the “typical group” by reviewing 153 patients with schizophrenia who were hospitalized in April 1940 at Kyoto University Hospital. Of these 153 patients, 68 were categorized into the atypical group (Table 1). Within the atypical group, acute or subacute onset occurred in 81%, acute‐phase lethality in 8%, complete recovery in 48%, and remission and recurrence in 32%. No patient in the typical schizophrenia group showed acute onset, lethality in the acute phase, or complete recovery during the observation period of 2 years. Eight representative atypical cases were described in Mitsuda's original paper (Table S3).

**Table 1:** Mitsuda's comparison of clinical courses and outcomes between the “typical group” and “atypical group” of schizophrenia patients

	Typical group (%)	Atypical group (%)
Acute onset	0	49
Subacute inset	21	32
Lethal in acute phase	0	8
Recovery	0	48
Remission and recurrence	0	32

Regarding atypical group, if the patients survived the acute phase, they recovered fully without sequelae. Mitsuda stated the atypical group might be caused somatically by parts of the body other than the brain in the first report.

Stauder's lethal catatonia syndrome featured acute onset psychosis with abnormal movement, cyanosis, fluctuation of autonomic functions, and lethal outcomes. A summary of the Stauder's cases presented in the original report is shown in Table S4. Psychosis, akinetic and hyperkinetic symptoms, mood fluctuation, consciousness and language disturbances, and family histories of various mental disorders were commonly recorded in both Mitsuda's and Stauder's patients. The mean age was in the early twenties, and the ratio of men to women was 1:1 in the populations. Mitsuda's atypical group had less frequent autonomic failure than Stauder's catatonia syndrome and might involve a broader spectrum.

Anti‐NMDAR encephalitis patients have characteristic combinations of neuropsychiatric symptoms with or without teratoma. With unique presentations, clinical diagnostic criteria for anti‐NMDAR encephalitis have been proposed.^7^ Our historical review lacked evidence of laboratory tests, teratomas or antibodies, and conclusive diagnoses could not be made retrospectively among the groups. However, Mitsuda's patients shown in Table S3 and Stauder's patients shown in Table S4 had at least four characteristic combinations of six major symptoms of anti‐NMDAR encephalitis.

Atypical psychoses as secondary or organic diseases have been discussed in relation to the borderland of epilepsy and the analysis of endocrinological functions during the mid‐twentieth century. We proposed that patients who were diagnosed with atypical psychoses in the twentieth century might include some of those with anti‐NMDAR encephalitis.

In a retrospective study, most patients with atypical psychoses could be reclassified according to the ICD‐10 standards as having acute polymorphic psychotic disorder with or without symptoms of schizophrenia, but some patients met the criteria for schizophrenia due to the longer duration of the episodes.^8^ Anti‐NMDAR antibodies have been detected among some patients with first‐episode psychosis who were initially diagnosed with schizophrenia with atypical presentations.^9^ The continuous spectrum of anti‐NMDAR encephalitis should not be restricted to patients with severe respiratory dysregulation.

Recently, “autoimmune psychosis” has been suggested as an emergent diagnostic category for isolated psychotic presentations of suspected autoimmune origin.^10^ The epidemiology of autoimmune psychosis has not been elucidated. Patients with first‐episode psychosis who initially exhibit an “atypical psychoses” phenotype should undergo cerebrospinal fluid analysis to determine whether antibodies against neuronal cell surface or synaptic receptors are present to rule out a possible diagnosis of autoimmune psychosis.

## Disclosure statement

The authors declare no conflict of interest.

## Supporting information


**Table S1**. Chronological table of the concept “atypical psychoses” in Japan.
**Table S2**. The comparison between atypical psychoses during mid‐twentieth century and modern anti‐NMDA receptor encephalitis.
**Table S3**. Summary of Mitsuda's patients with catatonia variants in the atypical group.
**Table S4**. Summary of Stauder's patients.
