# Supplementary material for: “Atypical psychoses” and anti‐NMDA receptor encephalitis: A review of literature in the mid‐twentieth century
Source: Psychiatry Clin Neurosci. 2021 Dec 23;76(2):62–3. doi: 10.1111/pcn.13317 (PMC9303716; doi:10.1111/pcn.13317)
Supplement: Supplementary file 1 — Table S1. Chronological table of the concept “atypical psychoses” in Japan. Table S2. The comparison between atypical psychoses during mid‐twentieth century and modern anti‐NMDA receptor encephalitis. Table S3. Summary of Mitsuda's patients with catatonia variants in the atypical group. Table S4. Summary of Stauder's patients. [file PCN-76-62-s001.docx]

**Table S1. Chronological table of the concept “atypical psychoses” in Japan.**

| Author | Year | Content | Reference |
| --- | --- | --- | --- |
| Mitsuda | 1942 | Atypical group of schizophrenia | 1) |
| Mitsuda | 1950 | Atypical psychoses | 2) |
| Sawa | 1957 | Epileptogenic factors in atypical psychoses | 3) |
| Kurosawa | 1961 | Similarity and difference between Mitsuda's atypical psychoses and the concepts by Leonhard, Pauleikoff, Ey | 4) |
| Hatotani | 1962 | The common clinical features of atypical psychoses | 5) |
| Mitsuda | 1965 | Expansion to a more comprehensive concept | 6) |

Mitsuda’s “atypical group” was first termed “atypical psychoses” in a case report of a family written in 1950. The symposium on “atypical psychoses” held in 1961 under the auspices of the Japanese Neuropsychiatric Association confirmed the widely known features as follows: (1) acute onset, sometimes following somatic or psychological stress (overwork, infection, pregnancy, or menstruation); (2) a favorable monophasic or polyphasic course without personality sequelae; (3) clouding of consciousness and emotional and psychomotor disturbances with vivid hallucinations; and (4) a reality-oriented premorbid character different from that of schizophrenia patients. Later, the term “atypical psychoses” merged an international consensus, which was led by German and French psychiatrists, including Kleist, Leonhard, Pauleikhoff and Ey, and was expanded to a more comprehensive concept by Mitsuda himself.

References

1. Mitsuda H. Klinisch-erbbiologische Untersuchung der Schizophrenie. *Psychiat Neurol Jap.* 1942; **46**: 297–362 (in Japanese).
2. Mitsuda H. On a pedigree of atypical psychoses. *Folia Psychiatr Neurol Jpn.* 1950; **4**: 115–122.
3. Sawa M. Epileptogenic factors in atypical endogenous psychoses. *Psychiat Neurol Jap.* 1957; **59**: 73–111 (in Japanese).
4. Kurosawa R. The concept and clinical practice of atypical psychoses. *Clin Psychiatry.* 1961; **3**: 959–965 (in Japanese).
5. Hatotani N, Ishida C, Yura R, Maeda M, Kato Y, Nomura J. Psycho-physiological studies of atypical psychoses-endocrinological aspect of periodic psychoses. *Folia Psychiatr Neurol Jpn.* 1962; **16**: 248–292.
6. Mitsuda H. The concept of "atypical psychoses" from the aspect of clinical genetics. *Acta Psychiatr Scand*. 1965; **41**: 372–377.

**Table S2.** **The comparison between atypical psychoses during mid-twentieth century and modern anti-NMDA receptor encephalitis.**

|  | Atypical psychoses | References | Anti-NMDA receptor encephalitis | References |
| --- | --- | --- | --- | --- |
| Onset | Acute | 1) | Acute | 2) |
| Lethality | 8% |  | 10% |  |
| Substantial improvement | 80% |  | 81% |  |
| Recurrence | 32% |  | 12% |  |
| Spontaneous remission | + |  | + |  |
| Trigger | Menstruation, pregnancy, delivery | 3) | Teratoma | 4) |
|  | Infection |  | Infection (especially herpes simplex encephalitis) | 5) |
|  | Psychological stress |  | Case report: psychological stress | 6) |
| Abnormal electroencephalogram | Enlarged fluctuation of irregulate serrate form* | 7) | Extreme delta brush | 8) |
|  | Seesaw phenomenon* | 9) | Slow waves | 10) |
|  | Evoked paroxysmal spike and wave complex by Metrazol | 11) | Epileptic discharge |  |
| Treatment | Steroid** | 12,13) | Immunosuppresive therapy | 2) |
|  | Electroconvulsive therapy*** | 14) | Case series: electroconvulsive therapy | 15,16) |
| Genetic susceptibility | + | 1) | + | 17) |

*The “enlarged fluctuation of irregulate serrate form” means low voltage fast activities on delta and theta waves during sleep electroencephalogram. The “seesaw phenomenon” means negative correlation between slow waves bursts and coma/intermittent periods.

**Corticosteroid treatment of patients with atypical psychoses has been reported in several case reports. A case series of lethal catatonia syndrome as a severe group of atypical psychoses reported 3 patients treated with prednisolone out of a total of 6 presented patients. In that report, an autopsy of a patient not treated with prednisolone revealed lymphocyte infiltration of the pia mater. The authors of the report referenced the paper, which reported the effectiveness of adrenal cortex extract for acute-onset fatal psychosis in 7 representative patients, published in 1946.

***Kirov K reported that electroconvulsive therapy was performed a total of 179 times for 456 episodes in 98 patients with atypical phasic psychosis. Remission occurred in 140 of the 179 instances in which electroconvulsive therapy was used (78%). Spontaneous remission without any treatment occurred 46 times out of a total 427 episodes (11%).

References

1. Mitsuda H. Klinisch-erbbiologische Untersuchung der Schizophrenie. *Psychiat Neurol Jap.* 1942; **46**: 297–362 (in Japanese).
2. Titulaer MJ, McCracken L, Gabilondo I, et al. Treatment and prognostic factors for long-term outcome in patients with anti-NMDA receptor encephalitis: an observational cohort study. *Lancet Neurol.* 2013; **12**: 157–65.
3. Hatotani N, Ishida C, Yura R, Maeda M, Kato Y, Nomura J. Psycho-physiological studies of atypical psychoses-endocrinological aspect of periodic psychoses. *Folia Psychiatr Neurol Jpn.* 1962; **16**: 248–292.
4. Dalmau J, Tüzün E, Wu HY, et al. Paraneoplastic anti–*N*-methyl-D-aspartate (NMDA)-receptor encephalitis associated with ovarian teratoma. *Ann Neurol*. 2007; **61**: 25–36.
5. Dalmau J, Armangué T, Planagumà J, et al. An update on anti-NMDA receptor encephalitis for neurologists and psychiatrists: mechanisms and models. *Lancet Neurol.* 2019; **18**: 1045–1057.
6. Obi CA, Thompson E, Mordukhaev L, Khan I, Zhang NJ. Anti-*N*-methyl-D-aspartate receptor encephalitis triggered by emotional stress. *Proc Bayl Univ Med Cent.* 2019; **32**: 605–606.
7. Sato T. Clinical encephalogram of atypical psychoses. *Clin Psychiatry.* 1961; **3**: 977–992 (in Japanese).
8. Schmitt SE, Pargeon K, Frechette ES, Hirsch LJ, Dalmau J, Friedman D. Extreme delta brush. A unique EEG pattern in adults with anti-NMDA receptor encephalitis. *Neurology.* 2012; **79**: 1094–1100.
9. Kimura B. Längsschnittliche Untersuchungen über Korrelation von EEG-Befund mit klinischen Bildern atypischer endogener Psychosen. *Psychiat Neurol Jap*. 1967; **69**: 1237–1259 (in Japanese).
10. Gillinder L, Warren N, Hartel G, Dionisio S, O’Gorman C. EEG findings in NMDA encephalitis —a systematic review. *Seisure.* 2019; **65**: 20–24.
11. Sawa M. Epileptogenic factors in atypical endogenous psychoses. *Psychiat Neurol Jap.* 1957; **59**: 73–111 (in Japanese).
12. Shulack MNR. Exhaustion syndrome in excited psychotic patients. *Am J Psychiatry.* 1946; **102**: 466–472.
13. Miyoshi K, Deguchi T, Honda S, Takeda A, Matsuoka T. Febrile episode and catatonia. — the so-called fatal catatonia and its related conditions. *Psychiat Neurol Jap.* 1968; **70**; 52–65 (in Japanese).
14. Kirov K. Untersuchung über die Behandlung atypischer phasischer Psychosen. *Psychiat Neurol Med Psychol Leipzig*. 1972; **24**: 160–165 (in German).
15. Tanguturi YC, Cundiff AW, Fuchs C. anti–*N*-methyl-D-aspartate receptor encephalitis an electroconvulsive therapy. Literature review and future directions. *Child Adolesc Psychiatric Clin N Am.* 2019; **28**: 79–89.
16. Kanbayashi T, Tsutsui K, Tanaka K et al. Anti-NMDA encephalitis in psychiatry; malignant catatonia, atypical psychosis and ECT. *Clin Neurol.* 2014; **54**: 1103–1106 (in Japanese).
17. Shy Y, Guo J, Ma X et al. anti–*N*-methyl-D-aspartate receptor (NMDAR) encephalitis is associated with *IRF7*, *BANK1* and *TBX21* polymorphisms in two populations. *Eur J Neurol.* 2021; **28**: 595–601.

**Table S3. Summary of Mitsuda’s patients with catatonia variant of atypical group.**

| Patient | 1 | 2 | 3 | 4 | 5 | 6 | 7 | 8 |
| --- | --- | --- | --- | --- | --- | --- | --- | --- |
| Onset age | 14 | 23, 32, 36 | 13, 14, 16, 18 | 15, 16, 17 | 16, 18 | 17, 18 | 21, 23, 26 | 21 |
| Acute onset | + | + | + | + | + | + | + | + |
| Course | 1 month long duration | Several months long | 2-3 months long | Several weeks to 1 month long | 10 days to 2 weeks long | 1 month long | 10 days to 5 months long | 10 days long |
| Psychiatric behavior or cognitive dysfunction | God presence sense, depression, fear | Insomnia, fox possession, fear, mania, agitation | Fear, mania, insomnia | Religious delusions, mania, agitation | Amnesia childishness, ictal laughter | Mania | Mania, anger, amnesia | Mania, depressive |
| Speech dysfunction | Mutism, scream | Mutism, scream | Logorrhea | Roaring, mutism | Logorrhea | Mutism |  | Logorrhea |
| Seizures | Several times/day |  |  |  |  |  |  |  |
| Movement disorder | + | + | + | + | + | + | + | + |
| Impaired consciousness | Coma | Confusion | Confusion, coma | + | + | Confusion | + | + |
| Dysautonomia |  |  |  |  |  |  |  |  |
| Teratoma |  |  |  |  | - |  |  |  |
| Outcome | Recovery | Slight sequelae | Recovery | Recovery | Recovery | Recovery | Recovery | Recovery |
| Number of 6 major symptoms of anti-NMDAR encephalitis | 5 | 4 | 4 | 4 | 4 | 4 | 4 | 4 |

Among the eight patients, four were women, with ages ranging from 14 to 26 (mean 21) years. All patients had acute onset and recovered after each episode. Six patients had remission and recurrence. The patients presented at least four of six major symptoms of anti-NMDAR encephalitis.

**Table S4. Summary of Stauder’s patients with acute lethal catatonia.**

| Patient | 1 | 2 | 3 | 4 |
| --- | --- | --- | --- | --- |
| Onset age | 23 | 25 | 19 | 20 |
| Acute onset | + | + | + | + |
| Course | 4 days | 10 days | 30 days | 3 weeks |
| Psychiatric and cognitive dysfunction | Insomnia, anorexia, fear,  violent behavior | Suicide attempt, violence, insomnia | Delusion, auditory hallucination | Delusion, insomnia |
| Speech dysfunction | Mutism | Incoherence, indirectness, logorrhea | Incoherence, mutism | Screaming |
| Seizures |  | + | + |  |
| Movement disorder | Catatonia | Repetitive movements, grimacing | Repetitive movements, catatonia | Catatonia |
| Impaired consciousness | Confusion | + | + | + |
| Dysautonomia/ hypoventilation | Fever, cyanosis, urinary retention | Fever, cyanosis | Cyanosis | Oxygen need, arrhythmia, hypertension |
| Teratoma |  |  |  |  |
| Outcome | Death | Death | Death | Death |
| Number of positives of 6 major symptoms of anti-NMDAR encephalitis | 5 | 6 | 6 | 5 |
| Neuroleptic drugs | Scopolamine | Scopolamine, laudanum |  |  |

Among the four patients, two were women, with ages ranging from 19 to 25 (mean 22) years. All the patients had acute onset, autonomic dysfunction and hypoventilation and died within 30 days after onset. The patients presented at least five of six major symptoms of anti-NMDAR encephalitis, including psychiatric behavior, speech dysfunction, seizures, movement disorder, consciousness disturbance, and dysautonomia.
